# A Review of Deep Learning-Based Contactless Heart Rate Measurement Methods

**DOI:** 10.3390/s21113719

**Published:** 2021-05-27

**Authors:** Aoxin Ni, Arian Azarang, Nasser Kehtarnavaz

**Affiliations:** Department of Electrical and Computer Engineering, The University of Texas at Dallas, Richardson, TX 75080, USA; azarang@utdallas.edu (A.A.); kehtar@utdallas.edu (N.K.)

**Keywords:** remote PPG, heart rate measurement methods, deep learning

## Abstract

The interest in contactless or remote heart rate measurement has been steadily growing in healthcare and sports applications. Contactless methods involve the utilization of a video camera and image processing algorithms. Recently, deep learning methods have been used to improve the performance of conventional contactless methods for heart rate measurement. After providing a review of the related literature, a comparison of the deep learning methods whose codes are publicly available is conducted in this paper. The public domain UBFC dataset is used to compare the performance of these deep learning methods for heart rate measurement. The results obtained show that the deep learning method PhysNet generates the best heart rate measurement outcome among these methods, with a mean absolute error value of 2.57 beats per minute and a mean square error value of 7.56 beats per minute.

## 1. Introduction

Physiological measurements are widely used to determine a person’s health condition [1,2,3,4,5,6]. Photoplethysmography (PPG) is a physiological measurement method that is used to detect volumetric changes in blood in vessels beneath the skin [1]. Medical devices based on PPG have been introduced to measure different physiological measurements including heart rate (HR), respiratory rate, heart rate variability (HRV), oxyhemoglobin saturation, and blood pressure [2,3,4,5,6]. Due to its low cost and non-invasive nature, PPG is utilized in many devices such as finger pulse oximeters, sports bands, and wearable sensors.

PPG-based physiological measurements can be categorized into two types: contact-based and contactless. Several survey articles have appeared in the literature on contact-based PPG methods as well as on contactless PPG methods. Contact-based methods deploy a light source and a photodetector. On the other hand, contactless methods deploy a video camera to measure the PPG signal. The previous survey articles mostly addressed conventional signal processing approaches. The recently developed deep learning-based methods have shown more promising results compared to the conventional methods. The focus of this review paper is thus placed on deep learning-based contactless methods for heart rate measurement.

**Figure 1:**
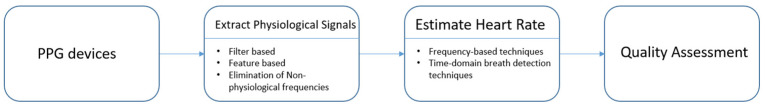
PPG signal processing framework.

A common practice in the medical field to measure the heart rate is ECG or electrocardiography [7,8], where voltage changes in the heart electrical activity are detected using electrodes placed on the skin. In general, ECG provides a more reliable heart rate measurement compared to PPG [9,10]. Hence, ECG is often used as the reference for evaluation of PPG methods [7,8,9,10]. Typically, 10 electrodes of the ECG machine are attached to different parts of the body including the wrist and ankle. Different from ECG, PPG-based medical devices possess differing sensor shapes placed on different parts of the body such as rings, earpieces, and bands [7,11,12,13,14,15,16], and they all use a light source and a photodetector to detect the PPG signal with signal processing, see Figure 1. The signal processing is for the purpose of processing the reflected optical signal from the skin [1].

Early research in this field concentrated on obtaining the PPG signal and ways to perform pulse wave analysis [17]. A comparison between ECG and PPG is discussed in [18,19]. There are survey papers covering different PPG applications that involve the use of wearable devices [20,21], atrial fibrillation detection [22], and blood pressure monitoring [23]. Papers have also been published which used deep learning for contact-based PPG, e.g., [24,25,26,27]. The previous survey papers on contact-based PPG methods are listed in Table 1.

Although contact-based PPG methods are non-invasive, they can be restrictive due to the requirement of their contact with the skin. Contact-based methods can be irritating or distracting in some situations, for example, for newborn infants [28,29,30,31]. When a less restrictive approach is desired, contactless PPG methods are considered. The use of contactless PPG methods or remote PPG (rPPG) methods has been growing in recent years [32,33,34,35,36].

**Figure 2:**
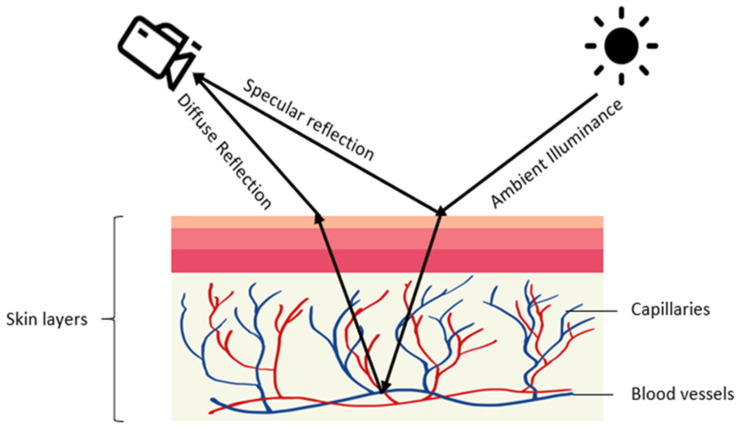
Illustration of rPPG generation: diffused and specular reflections of ambient illuminance are captured by a camera with the diffused reflection indicating volumetric changes in blood vessels.

**Figure 3:**
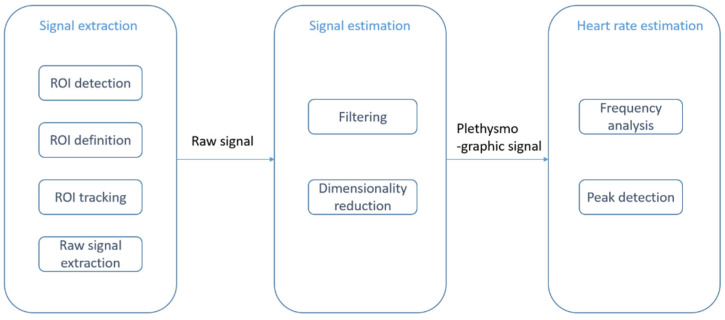
rPPG or contactless PPG image processing framework: signal extraction step (ROI detection and tracking), signal estimation step (filtering and dimensionality reduction), and heart rate estimation step (frequency analysis and peak detection).

Contactless PPG methods usually utilize a video camera to capture images which are then processed by image processing algorithms [32,33,34,35,36]. The physics of rPPG is similar to contact-based PPG. In rPPG methods, the light-emitting diode in contact-based PPG methods is replaced with ambient illuminance, and the photodetector is replaced with a video camera, see Figure 2. The light reaching the camera sensor can be separated into static (DC) and dynamic (AC) components. The DC component corresponds to static elements including tissue, bone, and static blood, while the AC component corresponds to the variations in light absorption due to arterial blood volume changes. Figure 3 provides an illustration of the image processing framework in rPPG methods. The common image processing steps involved in the framework are illustrated in this figure. In the signal extraction part of the framework, a region of interest (ROI), normally on the face, is extracted.

In earlier studies, video images from motionless faces were considered [37,38,39]. Several papers relate to exercising situations [40,41,42,43,44]. ROI detection and ROI tracking constitute two major image processing parts of the framework. The Viola and Jones (VJ) algorithm [45] is often used to detect face areas [46,47,48,49]. As an example of prior work on skin detection, a neural network classifier was used to detect skin-like pixels in [50]. In the signal estimation part, a bandpass filter is applied to eliminate undesired frequency components. A common choice for the frequency band is [0.7 Hz, 4 Hz], which corresponds to an HR between 42 and 240 beats per minute (bpm) [50,51,52,53]. To separate a signal into uncorrelated components and to reduce dimensionality, independent component analysis (ICA) was utilized in [54,55,56,57] and principal component analysis (PCA) was utilized in [38,39,40,58,59]. In the heart rate estimation module, the dimensionality-reduced data will be mapped to certain levels using frequency analysis or peak detection methods. The survey papers on rPPG methods that have already appeared in the literature are listed in Table 2. These survey papers provide comparisons with contact-based PPG methods.

There are challenges in rPPG which include subject motion and ambient lighting variations [60,61,62]. Due to the success of deep learning in many computer vision and speech processing applications [63,64,65], deep learning methods have been considered for rPPG to deal with its challenges, for example, [44,49]. In deep learning methods, feature extraction and classification are carried out together within one network structure. The required datasets for deep learning models are collected using RGB cameras. As noted earlier, the focus of this review is on deep learning-based contactless heart rate measurement methods.

**Table 2:** Survey papers on conventional contactless methods previously reported in the literature.

Emphasis	Ref	Year	Task
Contactless	[66]	2018	Provides typical components of rPPG and notes the main challenges; groups published studies by their choice of algorithm.
Contactless	[67]	2012	Covers three main stages of monitoring physiological measurements based on photoplethysmographic imaging: image acquisition, data collection, and parameter extraction.
Contactless and contact	[68]	2016	States review of contact-based PPG and its limitations; introduces research activities on wearable and non-contact PPG.
Contactless and contact	[69]	2009	Reviews photoplethysmographic measurement techniques from contact sensing placement to non-contact sensing placement, and from point measurement to imaging measurement.
Contactless newborn infants	[28]	2013	Investigates the feasibility of camera-based PPG for contactless HR monitoring in newborn infants with ambient light.
Contactless newborn infants	[30]	2016	Comparative analysis to benchmark state-of-the-art video and image-guided noninvasive pulse rate (PR) detection.
Contactless and contact	[70]	2017	Heart rate measurement using facial videos based on photoplethysmography and ballistocardiography.
Contactless and contact	[71]	2014	Covers methods of non-contact HR measurement with capacitively coupled ECG, Doppler radar, optical vibrocardiography, thermal imaging, RGB camera, and HR from speech.
Contactless RR and contact	[72]	2011	Discusses respiration monitoring approaches (both contact and non-contact).
Contactless newborn infants	[31]	2019	Addresses HR measurement in babies.
Contactless	[73]	2019	Examines challenges associated with illumination variations and motion artifacts.
Contactless	[74]	2017	Covers HR measurement techniques including camera-based photoplethysmography, reflectance pulse oximetry, laser Doppler technology, capacitive sensors, piezoelectric sensors, electromyography, and a digital stethoscope.
Contactless Main challenges	[75]	2015	Covers issues in motion and ambient lighting tolerance, image optimization (including multi-spectral imaging), and region of interest optimization.

In essence, this paper provides a review of combinations of conventional and deep learning rPPG methods as well as end-to-end deep learning-based rPPG methods for heart rate measurement. More specifically, the deep learning-based methods for heart rate measurement are grouped into two main categories, and the ones whose codes are publicly available are compared by examining the same public domain dataset.

## 2. Contactless PPG Methods Based on Deep Learning

Previous works on deep learning-based contactless HR methods can be divided into two groups: combinations of conventional and deep learning methods, and end-to-end deep learning methods. In what follows, a review of these papers is provided. Later, in Section 3, the end-to-end deep learning methods whose codes are publicly available are compared by applying them to the same public domain dataset.

### 2.1. Combination of Conventional and Deep Learning Methods

Li et al. 2021 [76] presented multi-modal machine learning techniques related to heart diseases. From Figure 3, it can be seen that one or more components of the contactless HR framework can be achieved by using deep learning. These components include ROI detection and tracking, signal estimation, and HR estimation.

#### 2.1.1. Deep Learning Methods for Signal Estimation

Qiu et al. 2018 [77] developed a method called EVM-CNN. The pipeline of this method consists of three modules: face detection and tracking, feature extraction, and HR estimation. In the face detection and tracking module, 68 facial landmarks inside a bounding box are detected by using a regression local binary features-based approach [78]. Then, an ROI defined by eight points around the central part of a human face is automatically extracted and inputted into the next module. In the feature extraction module, spatial decomposition and temporal filtering are applied to obtain so-called feature images. The sequence of ROIs is down-sampled into several bands. The lowest bands are reshaped and concatenated into a new image. Three channels of this new image are transferred into the frequency domain; then, fast Fourier transform (FFT) is applied to remove the unwanted frequency bands. Finally, the bands are transferred back to the time domain by performing inverse FFT and merging into a feature image. In the HR estimation module, a convolutional neural network (CNN) is used to estimate HR from the feature image. The CNN used in this method has a simple structure with several convolution layers which uses depth-wise convolution and point-wise convolution to reduce the computational burden and model size.

**Figure 4:**
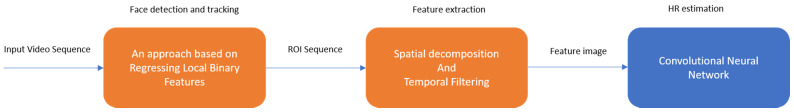
EVM-CNN modules.

As shown in Figure 4, in this method, the first two modules which are face detection/tracking and feature extraction are conventional rPPG approaches, whereas the HR estimation module uses deep learning to improve performance for HR estimation.

#### 2.1.2. Deep Learning Methods for Signal Extraction

Luguev et al. 2020 [79] established a framework which uses deep spatial-temporal networks for contactless HRV measurements from raw facial videos. In this method, a 3D convolutional neural network is used for pulse signal extraction. As for the computation of HRV features, conventional signal processing methods including frequency domain analysis and peak detection are used. More specifically, raw video sequences are inputted into the 3D-CNN without any skin segmentation. Several convolution operations with rectified linear units (ReLU) are used as activation functions together with pooling operations to produce spatiotemporal features. In the end, a pulse signal is generated by a channel-wise convolution operation. The mean absolute error is used as the loss function of the model.

**Figure 5:**
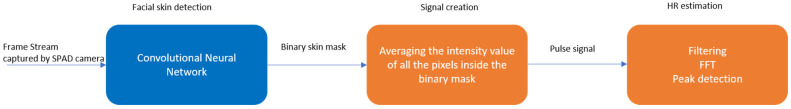
rPPG using SPAD camera.

Paracchini et al. 2020 [80] implemented rPPG based on a single-photon avalanche diode (SPAD) camera. This method combines deep learning and conventional signal processing to extract and examine the pulse signal. The main advantage of using a SPAD camera is its superior performance in dark environments compared with CCD or CMOS cameras. Its framework is shown in Figure 5. The signal extraction part has two components which are facial skin detection and signal creation. A U-shape network is then used to perform skin detection including all visible facial skin surface areas rather than a specific skin area. The output of the network is a binary skin mask. Then, a raw pulse signal is obtained by averaging the intensity values of all the pixels inside the binary mask. As for signal estimation, this is achieved by filtering, FFT, and peak detection. The experimental results include HR, respiration rate, and tachogram measurements.

In another work from Zhan et al. 2020 [81], the focus was placed on understanding the CNN-based PPG signal extraction. Four questions were addressed: (1) Does the CNN learn PPG, BCG, or a combination of both? (2) Can a finger oximeter be directly used as a reference for CNN training? (3) Does the CNN learn the spatial context information of the measured skin? (4) Is the CNN robust to motion, and how is this motion robustness achieved? To answer these four questions, a CNN-PPG framework and four experiments were designed. The results of these experiments indicate the availability of multiple convolutional kernels is necessary for a CNN to arrive at a flexible channel combination through the spatial operation but may not provide the same motion robustness as a multi-site measurement. Another conclusion reached is that the PPG-related prior knowledge may still be helpful for the CNN-based PPG extraction.

### 2.2. End-to-End Deep Learning Methods

In this section, end-to-end deep learning systems are stated which take video as the input and use different network architectures to generate a physiological signal as the output.

#### 2.2.1. VGG-Style CNN

Chen and Mcduff 2018 [82] developed an end-to-end method for video-based heart and breathing rates using a deep convolutional network named DeepPhys. To address the issue caused by subject motion, the proposed method uses a motion representation algorithm based on a skin reflection model. As a result, motions are captured more effectively. To guide the motion estimation, an attention mechanism using appearance information was designed. It was shown that the motion representation model and the attention mechanism used enable robust measurements under heterogeneous lighting and motions.

**Figure 6:**
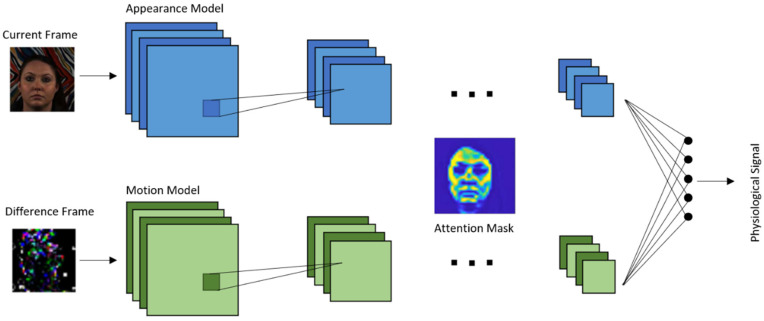
DeepPhys architecture.

The model is based on a VGG-style CNN for estimating the physiological signal derived under motion [83]. VGG is an object recognition model that supports up to 19 layers. Built as a deep CNN, VGG is shown to outperform baselines in many image processing tasks. Figure 6 illustrates the architecture of this end-to-end convolutional attention network. A current video frame at time t and a normalized difference between frames at t and t + 1 constitute the inputs to the appearance and motion models, respectively. The network learns spatial masks, which are shared between the models, and extracts features for recovering the blood volume pulse (BVP) and respiration signals.

Deep PPG proposed by Reiss et al. 2019 [84] addresses three shortcomings of the existing datasets. First is the dataset size. While the number of subjects can be considered as sufficient (8–24 participants in each dataset), the length of each session’s recording can be rather short. Second is the small numbers of activities. The publicly available datasets include data from only two–three different activities. Additionally, third is data recording in laboratory settings rather than in real-world environments.

**Figure 7:**
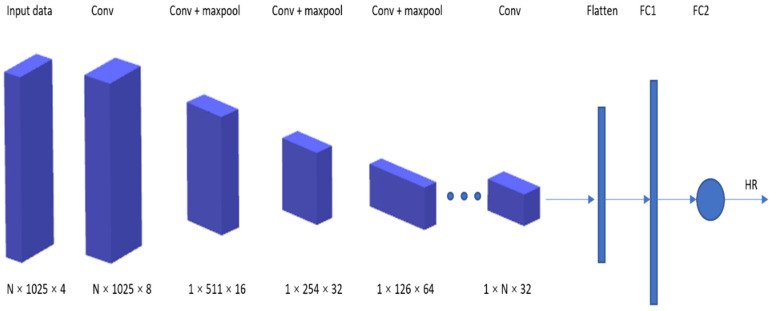
Deep PPG architecture.

A new dataset, called PPG-DaLiA [85], was thus introduced in this paper: a PPG dataset for motion compensation and heart rate estimation in daily living activities. Figure 7 illustrates the architecture of the VGG-like CNN used, where the time–frequency spectra of PPG signals are used as the input to estimate the heart rate.

#### 2.2.2. CNN-LSTM Network

Long short-term memory (LSTM) is a recurrent neural network (RNN) architecture which allows only process handling a single data point (such as images), but also an entire sequence of data points (such as speech or video). It has been previously used for various tasks such as connected handwriting recognition, speech recognition, and anomaly detection in network traffic [86,87,88].

rPPG signals are usually collected using a video camera with a limitation of being sensitive to multiple contributing factors, which include variation in skin tone, lighting condition, and facial structure. Meta-rPPG [89] is an end-to-end supervised learning approach which performs well when training data are abundant with a distribution that does not deviate too much from the testing data distribution. To cope with the unforeseeable changes during testing, a transductive meta-learner that takes unlabeled samples during testing for a self-supervised weight adjustment is used to provide fast adaptation to the changes. The network proposed in this paper is split into two parts: a feature extractor and an rPPG estimator modeled by a CNN and an LSTM network, respectively.

#### 2.2.3. 3D-CNN Network

A 3D convolutional neural network is a type of network with kernel sliding in three dimensions. 3D-CNN is shown to have better performance in spatiotemporal information learning than 2DCNN [90].

**Figure 8:**
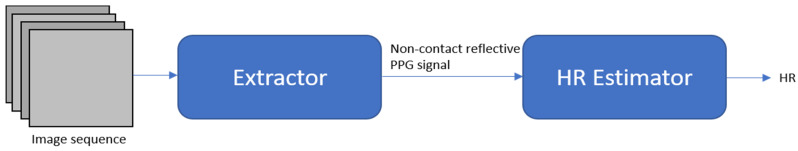
HR-CNN modules.

Špetlík et al. 2018 [46] proposed a two-step convolutional neural network to estimate the heart rate from a sequence of facial images, see Figure 8. The proposed architecture has two components: an extractor and an HR estimator. The extractor component is run over a temporal image sequence of faces. The signal is then fed to the HR estimator to predict the heart rate.

In the work from Yu et al. 2019 [91], a two-stage end-to-end method was proposed. This work deals with video compression loss and recovers the rPPG signal from highly compressed videos. It consists of two parts: (1) a spatiotemporal video enhancement network (STVEN) for video enhancement, and (2) an rPPG network (rPPGNet) for rPPG signal recovery. rPPGNet can work on its own for obtaining rPPG measurements. The STVEN network can be added and jointly trained to further boost the performance, particularly on highly compressed videos.

Another method from Yu et al. 2019 [92] provides the use of deep spatiotemporal networks for reconstructing precise rPPG signals from raw facial videos. With the constraint of trend consistency in ground truth pulse curves, this method is able to recover rPPG signals with accurate pulse peaks. The heartbeat peaks of the measured rPPG signal are located at the corresponding R peaks of the ground truth ECG signal.

To address the issue of a lack of training data, a heart track convolutional neural network was developed by Rerepelkina et al. 2020 [93] for remote video-based heart rate tracking. This learning-based method is trained on synthetic data to accurately estimate the heart rate in different conditions. Synthetic data do not include video and include only PPG curves. To select the most suitable parts of the face for pulse tracking at each particular moment, an attention mechanism is used.

**Figure 9:**
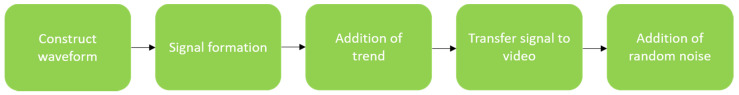
Process of generating synthetic data.

Similar to the previous methods, the method proposed by Bousefsaf et al. 2019 [94] also uses synthetic data. Figure 9 illustrates the process of how synthetic data are generated. A 3D-CNN classifier structure was developed for both extraction and classification of unprocessed video streams. The CNN acts as a feature extractor. Its final activations are fed into two dense layers (multilayer perceptron) that are used to classify the pulse rate. The network ensures concurrent mapping by producing a prediction for each local group of pixels.

Liu et al. 2020 [95] developed a lightweight rPPG estimation network, named DeeprPPG, based on spatiotemporal convolutions for utilization involving different types of input skin. To further boost the robustness, a spatiotemporal rPPG aggregation strategy was designed to adaptively aggregate rPPG signals from multiple skin regions into a final one. Extensive experimental studies were conducted to show its robustness when facing unseen skin regions in unseen scenarios. Table 3 lists the contactless HR methods that use deep learning.

## 3. Selected Deep Learning Models for Comparison

Among the deep learning-based rPPG methods, the codes for four methods are publicly available. In this section, a comparison of these methods is carried out. First, the architectures of these methods are stated in some detail.

### 3.1. STVEN-rPPGNet

**Figure 10:**
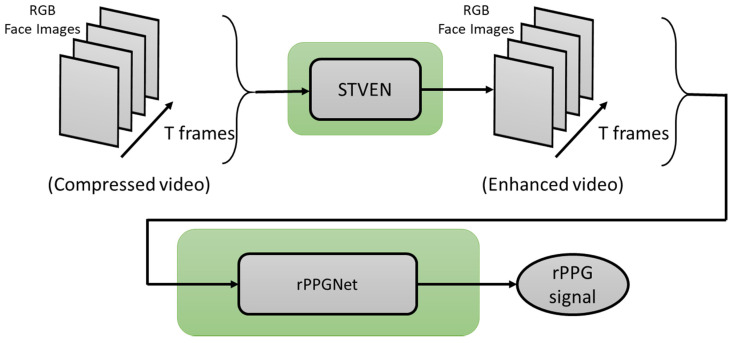
Architecture of STVEN-rPPGNet.

This deep learning-based method considers low-resolution input video clips to measure the heart rate. Its training occurs in two stages. The first stage involves a video enhancement network (called STVEN) whose output corresponds to spatially enhanced videos. The second stage involves a measurement network (called rPPGNet) whose output provides the heart rate. The measurement network rPPGNet is formed using a spatiotemporal convolutional network, a skin-based attention module, and a partition constraint module. The skin-based attention module selects skin regions. The partition constraint module enables an improved representation of the rPPG signal. An illustration of the two-stage architecture of STVEN-rPPGNet is shown in Figure 10.

### 3.2. IPPG-3D-CNN

In this method, the training phase is performed on synthetic data. That is, the pseudo-PPG video streams are formed by repeating waveforms, which are constructed by Fourier series approximation. In the testing phase, no pre-processing step, such as automatic face detection, is carried out. To synthesize video streams, the following steps are taken: (1) via Fourier series, a waveform model fitted to the rPPG waveform is generated, (2) based on the waveform in (1), a two-second signal is generated, (3) the signal is repeated to form a video stream, and (4) random noise at a specified noise level is added to each image of a video stream.

**Figure 11:**
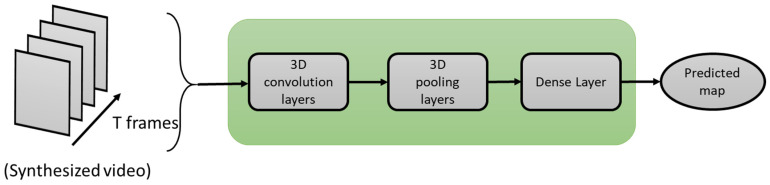
Architecture of iPPG-3D-CNN.

Then, video patches are fed into the network which are mapped to the targeted heart rate. By subtracting the average value, each video is centered around zero. Training is conducted by constantly adding 15,200 batches in duration (200 video patches in each of the 76 levels of heart rates). Thus, each batch changes the network parameters with respect to an input tensor of 15,200 × 25 × 25 × 60. An illustration of the architecture of this deep learning-based method is shown in Figure 11.

### 3.3. PhysNet

**Figure 12:**
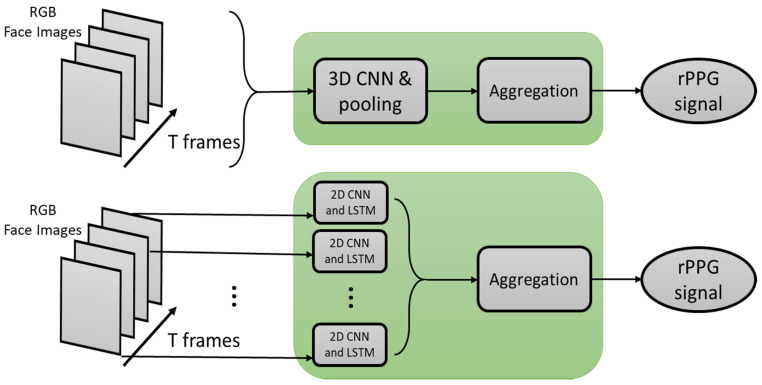
Architecture of PhysNet.

In this method, the RGB frames of the face are mapped into the rPPG domain directly without any pre- and post-processing step. In fact, the solution developed is an end-to-end one. The architecture of this deep neural network uses two different structures for training: (1) the first architecture maps the facial RGB frames into the rPPG signal via several convolution and pooling layers, and (2) the second architecture uses RNN processing units. The difference between the first and second structures is that T-frames are inputted to the first network structure at the same time, and 3D convolution layers are used in the second network structure by inputting one frame at a time. An illustration of the architecture of this deep learning-based method is depicted in Figure 12.

### 3.4. Meta-rPPG

The idea of using meta-learning for heart rate measurement from the rPPG signal is to fine-tune the parameters of a network for situations that are not covered in the training set. The architecture of this network consists of two parts: one part enables a fast adaptation process and the other part provides heart rate measurement. Its learning process involves the following: (1) extracting facial frames from video, and the face area is cropped with the region outside the face area set to zero to obtain facial landmarks, and (2) for each facial frame, the modified PPG signal, which is obtained by a small temporal offset, is used as the network target.

**Figure 13:**
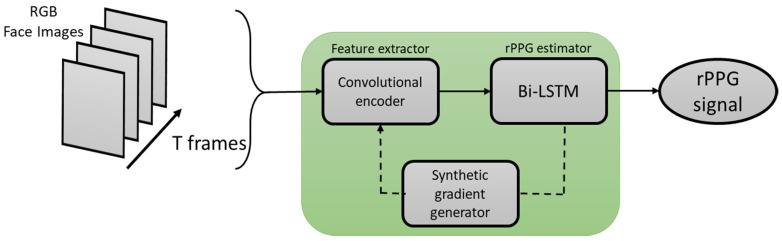
Architecture of Meta-rPPG.

The architecture of this network consists of three modules: convolutional encoder, rPPG estimator (with LSTM), and a synthetic gradient generator. During its inference mode, only the convolutional encoder and the rPPG estimator are used. The synthetic gradient estimator is utilized in its transductive mode. This network is designed to remove spatiotemporal features by modeling visual information using a deep convolutional encoder and then by modeling the PPG signal using Bi-LSTM. An illustration of the architecture of this deep learning-based method is provided in Figure 13.

## 4. Comparison Results and Discussion

This subsection demonstrates the comparison results of the above four algorithms whose codes are publicly available for the purpose of measuring the heart rate. The performance of these four algorithms is found in terms of bpm.

### 4.1. Dataset

The UBFC database [96] is used here to train and test the above four methods. This database consists of 37 uncompressed videos with a resolution of 640 × 480 in 8-bit RGB format. Each video corresponds to a specific subject. The ground truth value of the video data is PPG waveform (magnitude and time) along with heart rates recorded with a pulse oximeter. There is no need to perform any pre-processing on this database. Ten randomly selected subjects were used for our test set, and the rest were used for the training set.

### 4.2. Experimental Setup

In the studies conducted in [78,91,97,98], it was shown that the deep learning methods performed better than the conventional methods. Hence, the focus of the experimentation conducted here is placed on the above selected deep learning models. An overview of the architecture of the selected deep learning models is provided in Table 4.

The experiments for this study were conducted in one phase, where the above-mentioned dataset was divided into a training and a test set with no overlap. The image frames were extracted from the video clips using the MATLAB toolbox [99]. A region of interest (ROI) was then selected and cropped using the Viola–Jones algorithm [45] from the original image. One of the deep learning models required the skin map of the frames. The skin map of each image was extracted using the Bob package [100]. Finally, the extracted images and skin labels were then used to train and test the CNN-based pulse rate measurement algorithms. The outcomes of each of the four algorithms were assessed as a function of the mean square error (MSE) [101], mean absolute error (MAE) [102], and standard deviation (SD) [103]. To be fair in terms of objective metrics, the ratio of training and test sets was kept the same for all four selected deep models.

The metrics used for evaluation are stated next. As mentioned above, to quantify the performance of each deep learning method, the MSE and the MAE between the predicted heart rate and the ground truth were considered. The SDs of the reference heart rate and the predicted heart rate are also reported. The MSE and MAE were computed using the following equations:

(1) $$\;\mathrm{MSE}=\sqrt{\frac{1}{N}\sum_{i=1}^{N}\left|R_{i}-P_{i}\right|}$$

(2) $$\mathrm{MAE}=\frac{1}{N}\left|R_{i}-P_{i}\right|$$

where $R_{i}$ and $P_{i}$ denote the ground truth and predicted heart rates, respectively, and *N* is the total number of heartbeats.

### 4.3. Results and Discussion

The results obtained are reported in Table 5 for the test set. The reference value for each metric is placed in the last row of the table. In most cases, the PhysNet method performed better than the other deep learning methods in terms of the objective metrics. For instance, the MAE and MSE of subject 10 in PhysNet were found to be lower than the other methods.The same result was obtained for subject 5 as well. More specifically, the MAE of rPPGNet, 3D-CNN, PhysNet, and Meta-rPPG for subject 10 was found to be 3.14, 3.36, 2.60, and 3.67, respectively, whereas the MSE measure was found to be 10.74, 12.34, 7.63, and 14.60. The better performance of PhysNet is attributed to its architecture enabling the extraction of effective features from input frames.

The latency or computation time associated with each of the methods is also reported in Table 6 for a batch with a size of 64. As seen from this table, 3D-CNN takes only 0.74 s to predict the heart rate from 64 images. In other words, 3D-CNN runs the fastest among the four methods.

**Figure 14:**
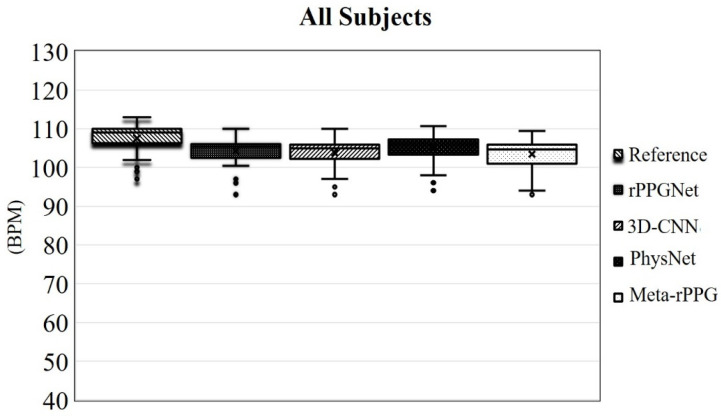
Averaged heart rate measurement of all the subjects in the test set. The vertical axis indicates the heart rate for each method in bpm. Each bar shows the mean and the standard deviation of a method. The first bar from the left indicates the reference.

**Figure 15:**
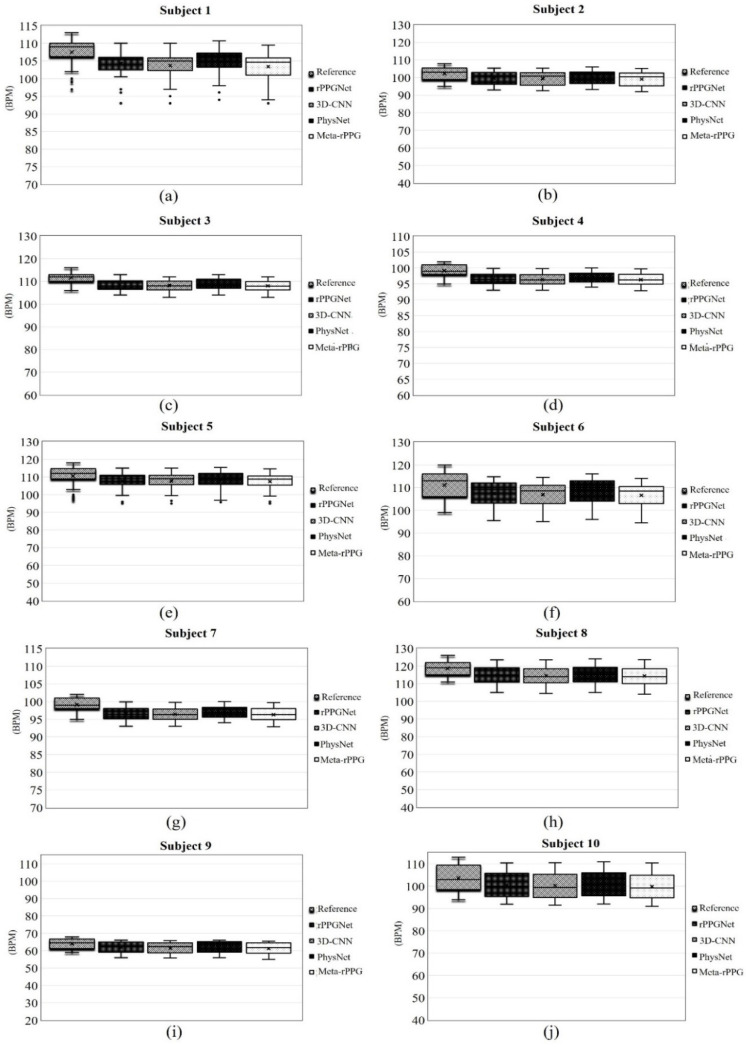
Heart rate bar charts of all the subjects in the test set for the four compared deep learning methods. Each part of figure (**a**–**j**) corresponds to one subject in the test set. The vertical axis indicates the heart rate in bpm. The mean and the standard deviation of each subject are specified in separate bar charts. In each chart, the first bar from the left indicates the reference for a subject.

To have an overall assessment of the four methods, the results were averaged for all the subjects. Figure 14 shows this outcome. As shown in this figure, the vertical axis corresponds to the range of the heart rate in bpm and the reference of the heart rate is denoted by the first bar from the left. From this figure, one can see that the average of the PhysNet method is closer to the reference. The results of individual subjects in the test set are shown in Figure 15. In this figure, the first bar from the left represents the reference. The legend associated with each bar is shown on the right side of the bar charts. By comparing the bar charts shown in this figure, one can see that PhysNet performs better than the other methods in terms of the mean and standard deviation. In other words, it provides the highest accuracy on average.

## 5. Conclusions

This paper has provided a comprehensive review of deep learning-based contactless heart rate measurement methods. First, an overview of contact-based PPG and contactless PPG methods was covered. Then, the review focus was placed on deep learning-based methods that have been introduced in the literature for heart rate measurement using rPPG. Among the deep learning-based contactless methods, four methods whose codes are publicly available were identified, and a comparison among these methods was conducted to see which one generates the highest accuracy for heart rate measurement by considering the same dataset across all four methods. Among these four methods, PhysNet was identified to provide the highest accuracy on average.

## Figures and Tables

**Table 1:** Previous survey papers on contact-based PPG methods.

Emphasis	Ref	Year	Task
Contact	[17]	2007	Basic principle of PPG operation, pulse wave analysis, clinical applications
Contact ECG and PPG	[18]	2018	Breathing rate (BR) estimation from ECG and PPG, BR algorithms and its assessment
Contact	[22]	2020	Approaches for PPG-based atrial fibrillation detection
Contact Wearable device	[20]	2019	PPG acquisition, HR estimation algorithms, developments on wrist PPG applications, biometric identification
Contact ECG and PPG	[19]	2012	Accuracy of pulse rate variability (PRV) as an estimate of HRV
Contact Wearable device	[21]	2018	Current developments and challenges of wearable PPG-based monitoring technologies
Contact Blood pressure	[23]	2015	Approaches involving PPG for continuous and non-invasive monitoring of blood pressure

**Table 3:** Deep learning-based contactless PPG methods.

Focus	Ref	Year	Feature	Dataset
End-to-end system Robust to illumination changes and subject’s motion	[46]	2018	A two-step convolutional neural network composed of an extractor and HR estimator	COHFACE PURE MAHNOB-HCI
Signal estimation enhancement	[77]	2019	Eulerian video magnification (EVM) to extract face color changes and using CNN to estimate heart rate	MMSE-HR
3D-CNN for signal extraction	[79]	2020	Using deep spatiotemporal networks for contactless HRV measurements from raw facial videos; employing data augmentation	MAHNOB-HCI
Single-photon camera	[80]	2020	Neural network for skin detection	N/A
Understanding of CNN-based PPG methods	[81]	2020	Analysis of CNN-based remote PPG to understand limitations and sensitivities	HNU PURE
End-to-end system Attention mechanism	[82]	2018	Robust measurement under heterogeneous lighting and motions	MAHNOB-HCI
End-to-end system Real-life conditions dataset	[84]	2019	Major shortcoming of existing datasets: dataset size, small number of activities, data recording in laboratory setting	PPG-DaLiA
Synthetic training data Attention mechanism	[93]	2020	CNN training with synthetic data to accurately estimate HR in different conditions	UBFC-RPPG MoLi-ppg-1 MoLi-ppg-2
Synthetic training data	[94]	2019	Automatic 3D-CNN training process with synthetic data with no image processing	UBFC-RPPG
End-to-end supervised learning approach Meta-learning	[89]	2017	Meta-rPPG for abundant training data with a distribution not deviating too much from distribution of testing data	MAHNOB-HCI UBFC-RPPG
Counter video compression loss	[91]	2019	STEVEN for video quality enhancement rPPGNet for signal recovery	MAHNOB-HCI
Spatiotemporal network	[92]	2019	Measuring rPPG signal from raw facial video; taking temporal context into account	MAHNOB-HCI
Spatiotemporal network	[95]	2020	Spatiotemporal convolution network, different types of input skin	MAHNOB-HCI PURE

**Table 4:** Overview of the selected network parameters.

Method	Network Architecture		
STVEN-rPPGNet	**Module**	**Layer**	**Kernel**
	STVEN	Convolution 1	3 × 3 × 7
		Convolution 2	3 × 4 × 4
		Convolution 3	4 × 4 × 4
		Spatiotemporal block	[3 × 3 × 3] × 6
		Deconvolution 1	4 × 4 × 4
		Deconvolution 2	1 × 4 × 4
		Deconvolution 3	1 × 7 × 7
	rPPGNet	Convolution 1	1 × 5 × 5
		Spatiotemporal block	[3 × 3 × 3] × 4
		Spatial global average pooling	1 × 16 × 16
		Deconvolution 1	1 × 1 × 1
iPPG-3 DCNN		Convolution 1	58 × 20 × 20
		Max pooling	2 × 2 × 2
		Dense	512
		Dense	76
PhysNet		Convolution 1	1 × 5 × 5
		Max pooling	1 × 2 × 2
		Convolution 2	3 × 3 × 3
		Convolution 3	3 × 3 × 3
		Spatial global average pooling	
		Convolution 4	1 × 1 × 1
Meta-rPPG		Convolution 1	3 × 3
		Convolution 2	3 × 3
		Convolution 3	3 × 3
	Convolutional Encoder	Convolution 4	3 × 3
		Convolution 5	3 × 3
		Average pooling	2 × 2
	rPPG Estimator	Bidirectional LSTM	---
		Linear	---
		Ordinal	---
	Synthetic Gradient Generator	Convolution 1	3 × 3
		Convolution 2	3 × 3
		Convolution 3	3 × 3
		Convolution 4	3 × 3

**Table 5:** Objective metrics for the four compared deep learning methods.

Subject #	Method	HR (bpm)		
		MAE	MSE	SD
Subject 1	rPPGNet	3.22	11.41	3.93
	3D-CNN	3.75	14.92	3.86
	PhysNet	2.53	7.31	3.96
	Meta-rPPG	4.09	17.67	3.95
Subject 2	rPPGNet	2.72	7.82	3.82
	3D-CNN	2.87	8.81	3.93
	PhysNet	2.25	5.47	3.79
	Meta-rPPG	3.18	10.71	4.01
Subject 3	rPPGNet	3.12	11.14	2.32
	3D-CNN	3.43	13.28	2.33
	PhysNet	2.74	8.74	2.42
	Meta-rPPG	3.63	14.78	2.36
Subject 4	rPPGNet	2.63	7.79	1.79
	3D-CNN	2.74	8.42	1.74
	PhysNet	2.14	5.48	1.75
	Meta-rPPG	2.83	8.96	1.77
Subject 5	rPPGNet	2.82	8.90	5.48
	3D-CNN	2.96	9.72	5.50
	PhysNet	2.38	6.66	5.54
	Meta-rPPG	3.22	11.37	5.48
Subject 6	rPPGNet	3.76	15.09	5.71
	3D-CNN	4.21	18.91	5.66
	PhysNet	2.93	9.26	5.95
	Meta-rPPG	4.56	22.34	5.63
Subject 7	rPPGNet	3.42	12.40	8.79
	3D-CNN	3.85	15.78	8.66
	PhysNet	2.91	9.04	8.94
	Meta-rPPG	4.01	17.02	8.72
Subject 8	rPPGNet	3.66	14.51	4.87
	3D-CNN	3.93	16.82	4.92
	PhysNet	3.18	11.21	4.92
	Meta-rPPG	4.20	19.07	4.96
Subject 9	rPPGNet	2.24	5.49	3.47
	3D-CNN	2.52	6.76	3.47
	PhysNet	2.04	4.76	3.55
	Meta-rPPG	2.78	8.13	3.58
Subject 10	rPPGNet	3.14	10.74	5.65
	3D-CNN	3.36	12.34	5.63
	PhysNet	2.60	7.63	5.77
	Meta-rPPG	3.67	14.60	5.62
Averaged across all subjects	rPPGNet	3.07	10.53	4.58
	3D-CNN	2.98	12.58	4.57
	PhysNet	2.57	7.56	4.66
	Meta-rPPG	3.62	14.47	4.61
Reference value		0	0	0

**Table 6:** Latency or computation time for the four compared deep learning methods.

Method	rPPGNet	3D-CNN	PhysNet	Meta-rPPG
Time	1.12 (s)	0.74 (s)	1.19 (s)	1.7 (s)

## Data Availability

Not applicable.
